# Impact of non‐CNS childhood cancer on resting‐state connectivity and its association with cognition

**DOI:** 10.1002/brb3.1931

**Published:** 2020-11-18

**Authors:** Janine S. Spitzhüttl, Martin Kronbichler, Lisa Kronbichler, Valentin Benzing, Valerie Siegwart, Manuela Pastore‐Wapp, Claus Kiefer, Nedelina Slavova, Michael Grotzer, Claudia M. Roebers, Maja Steinlin, Kurt Leibundgut, Regula Everts

**Affiliations:** ^1^ Department of Psychology University of Bern Bern Switzerland; ^2^ Neuropediatrics, Development and Rehabilitation University Children's Hospital Bern, and University of Bern Bern Switzerland; ^3^ Department of Pediatric Hematology and Oncology University Children's Hospital Bern University of Bern Bern Switzerland; ^4^ Centre for Cognitive Neuroscience and Department of Psychology University of Salzburg Salzburg Austria; ^5^ Neuroscience Institute Christian‐Doppler Medical Centre Paracelsus Medical University Salzburg Austria; ^6^ Department of Psychiatry, Psychotherapy and Psychosomatics Christian‐Doppler Medical Centre, Paracelsus Medical University Salzburg Austria; ^7^ Institute of Sport Science University of Bern Bern Switzerland; ^8^ Support Center for Advanced Neuroimaging (SCAN) Institute of Diagnostic and Interventional Neuroradiology, Inselspital Bern University Hospital, and University of Bern Bern Switzerland; ^9^ Department of Pediatric Oncology University Children's Hospital Zurich Zurich Switzerland

**Keywords:** childhood cancer survivors, cognitive late‐effects, non‐CNS childhood cancers, resting‐state networks, rs‐fMRI analysis

## Abstract

**Introduction:**

Non‐central nervous system cancer in childhood (non‐CNS CC) and its treatments pose a major threat to brain development, with implications for functional networks. Structural and functional alterations might underlie the cognitive late‐effects identified in survivors of non‐CNS CC. The present study evaluated resting‐state functional networks and their associations with cognition in a mixed sample of non‐CNS CC survivors (i.e., leukemia, lymphoma, and other non‐CNS solid tumors).

**Methods:**

Forty‐three patients (off‐therapy for at least 1 year and aged 7–16 years) were compared with 43 healthy controls matched for age and sex. High‐resolution T1‐weighted structural magnetic resonance and resting‐state functional magnetic resonance imaging were acquired. Executive functions, attention, processing speed, and memory were assessed outside the scanner.

**Results:**

Cognitive performance was within the normal range for both groups; however, patients after CNS‐directed therapy showed lower executive functions than controls. Seed‐based connectivity analyses revealed that patients exhibited stronger functional connectivity between fronto‐ and temporo‐parietal pathways and weaker connectivity between parietal‐cerebellar and temporal‐occipital pathways in the right hemisphere than controls. Functional hyperconnectivity was related to weaker memory performance in the patients' group.

**Conclusion:**

These data suggest that even in the absence of brain tumors, non‐CNS CC and its treatment can lead to persistent cerebral alterations in resting‐state network connectivity.

## Graphical abstract

Non‐CNS childhood cancer is associated with alterations in functional connectivity. In patients, we observed both hyper‐ and hypoconnectivity when compared with healthy controls, however, altered functional connectivity was found only in the right hemisphere. Fronto‐parietal long‐range connections are more likely to be altered than short‐range connections. Stronger functional connectivity is associated with weaker verbal memory performance in survivors of childhood cancer.

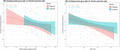

**Abbreviations**
AFNI: Analysis of Functional NeuroImages
ALL: acute lymphoblastic leukemia
BOLD: blood‐oxygen‐level‐dependent
CC: childhood cancer
CNS: central nervous system
IQ: intelligence quotient
MR: magnetic resonance
ROIs: regions of interest
rs‐fMR: resting‐state functional magnetic resonance imaging
WMTB‐C: working memory test battery for children

**Highlights**

- Non‐central nervous system childhood cancer (CC) is associated with alterations in functional connectivity.
- In patients, we observed both, hyper‐ and hypoconnectivity when compared with healthy controls.
- Altered functional connectivity was only found in the right hemisphere.
- Fronto‐parietal long‐range connections are more likely to be altered than short‐range connections.
- Stronger functional connectivity is associated with weaker memory performance in survivors of CC.

## 1 INTRODUCTION

Although the survival rates of those affected by childhood cancer (CC) in developed countries have greatly improved, CC is still the leading cause of death among childhood diseases (Arceci, 2016). The incidence of CC is 16:100,000 per year: it is highest among children aged 2 years, and the average age at the time of diagnosis is 6 years (Schindler et al., 2015). At this young age, dynamic processes of cortical brain development, which involve progressive and regressive gray and white matter changes such as myelination and synaptic pruning, are taking place (Casey et al., 2005; Gogtay et al., 2004). If undisturbed, these processes support the development of efficient and interconnected neural networks that meet our daily cognitive demands (Grayson & Fair, 2017).

Functional networks are already present in infancy but the functional organization (i.e., the functional coupling within a network) of sensory networks is known to occur much earlier than the functional organization of networks that are associated with higher‐order cognitive performance such as the dorsal attention, salience, and fronto‐parietal executive networks (Grayson & Fair, 2017). In contrast, network integration (i.e., functional coupling between networks) undergoes prolonged development, which continues until adulthood (Marek et al., 2015). It is suggested that pruning and myelination enable more efficient communication between regions (Grayson & Fair, 2017).

Disturbances to the development of networks not only occur in survivors of pediatric brain tumors (Chen et al., 2016; Na et al., 2018) but also affect children with cancers outside the CNS such as leukemia and lymphoma (Kesler et al., 2014, 2016). Leukemia and its potentially neurotoxic therapy might disrupt the functional and structural development of the brain (Hearps et al., 2017). Survivors of childhood bone and soft tissue sarcomas are prone to white matter alterations (Sleurs et al. 2018) and long‐term leukoencephalopathy possibly coming along with inflammation, changes in vasculature or axonal injury (Sleurs et al. 2019). Hence, network integration relying on long‐range connections that are refined and integrated later in the development process are expected to be particularly susceptible to the disease‐related effects of non‐CNS CC and its treatment.

Contemporary treatments for non‐CNS cancers include surgery, chemotherapy, and radiation therapy. Radiation therapy and chemotherapy (e.g., with methotrexate) are associated with direct harm to oligodendrocytes, axons, and microglia. As a consequence, demyelination, secondary immunologic reactions triggering inflammation, and microvascular alterations leading to blood vessel obstruction and necrosis may occur (Saykin et al., 2003). However, evidence suggests that even before any treatment begins, brain injury induced by the cancer itself (such as glial cell injury and demyelination) affects the developing brain (Cheung et al., 2018; Kesler et al., 2016; Patel et al., 2015).

Because cortical maturation is related to cognitive development, brain alterations caused by cancer and its treatment interfere with the development of cognitive abilities (Hearps et al., 2017). In fact, 40–60% of the children affected by acute lymphoblastic leukemia (ALL) and treated with chemotherapy were reported to experience cognitive late effects (van der Plas et al., 2015). Late maturing brain regions that undergo prolonged development, such as frontal lobe areas linked to executive functions, are particularly at risk (Gogtay et al., 2004; Walsh et al., 2015; Siegwart et al. 2020). Cognitive late‐effects are further apparent in specific core processing skills—particularly, processing speed and attention (Kahalley et al., 2013; Pierson et al., 2016; Wengenroth et al., 2015)—which in turn contribute to the development of higher‐order executive functions (Hearps et al., 2017; Patel et al., 2016). Executive functions enable the child to learn, integrate, and apply new abilities. Deficits in executive functions negatively impact the CC survivors’ academic and vocational career and quality of life (Hearps et al., 2017; Van Der Plas et al., 2018).

Non‐CNS CC survivors may exhibit alterations in functional networks that can be measured using resting‐state functional magnetic resonance imaging (rs‐fMRI; Hearps et al., 2017). Functional connectivity networks are examined by measuring the temporal coherence of spontaneous blood‐oxygen‐level‐dependent (BOLD) activity of anatomically separated brain regions during a state of quiet wakefulness when not performing any goal‐directed tasks (Biswal et al., 1995). Examining functional connectivity may be particularly valuable for investigating the neural underpinnings of higher‐order cognitive processes (i.e., executive functions), since these functions are assumed to rely on the interplay of spatially distributed regions at rest. Indeed, research has demonstrated associations between alterations in functional network connectivity and cognitive performance in children with non‐CNS cancer (Carey et al., 2008; Kesler et al., 2016; Kesler et al., 2010; Morioka et al., 2013).

There are different approaches to analyzing rs‐fMRI such as the seed‐based method (correlation of a seed region with all other voxels) or the independent component analysis (ICA, presentation of various independent components based on the BOLD signals). The seed‐based approach is useful for the detailed analysis of a particular region of interest, whereas the ICA clearly identifies all the independent networks (Lee et al., 2013). Both of these methods are able to successfully identify resting‐state networks (Rosazza et al., 2012). Using a seed‐based approach, Kesler et al. (2014) showed that resting‐state networks in 15 survivors of ALL exhibited an altered connectivity pattern (both hyper‐ and hypoconnectivity), compared with controls. Functional hypoconnectivity was associated with poorer IQ and visual color processing, and younger age at time of diagnosis.

Kesler et al. (2014) provides first important insights into functional connectivity and its relationship with executive functions in non‐CNS CC survivors. In adults, nonirradiated childhood leukemia survivors show altered brain connectivity, which was linked with cognitive flexibility (Billiet et al. 2018). We aimed to expand these investigations and examined the brain‐behavior relationship in CC survivors years after termination of treatment by (a) examining a relatively large sample of non‐CNS CC survivors and a group of age‐ and sex‐matched controls using a seed‐based resting‐state functional connectivity approach in an exploratory study design. Given that cancer treatments with CNS‐directed therapy show a higher degree of neurotoxicity than non‐CNS‐directed therapy (Krull et al., 2018; Zając‐Spychała et al., 2017), we further (b) examined cognition and functional connectivity in respect to the therapy approach (non‐CNS‐directed vs. CNS‐directed therapy). In addition, we (c) hypothesized that functional connectivity is associated with cognitive performance. Thereby, we focused on cognitive domains that have been typically described as vulnerable in non‐CNS CC survivors, such as executive functions, processing speed, attention, and memory. Furthermore, we investigated (d) the effects of age, age at diagnosis, time since treatment, and socioeconomic status on functional networks at rest.

## 2 MATERIAL AND METHODS

### 2.1 Participants

The present study used data collected as part of the Brainfit‐Study, a multidisciplinary clinical research program aiming to investigate the effect of cognitive and physical training in pediatric CC survivors (Benzing et al., 2018). Data presented in this study was acquired at the first assessment which took place before the cognitive and physical training and hence before randomization to a treatment arm. The Brainfit‐Study was approved by the ethics committee of the cantons of Bern and Zurich (Switzerland). The present study concerned a subsample of the Brainfit‐Study collective, namely children and adolescents who had survived non‐CNS CC.

#### 2.1.1 Non‐CNS CC survivors

All patients were recruited from the Swiss Childhood Cancer Registry and had been treated in one of the two pediatric university hospitals in Switzerland (Bern or Zurich). Inclusion criteria for the Brainfit‐Study were as follows: (a) diagnosis of noncentral nervous system cancer in the past 10 years; (b) aged between 7 and 16 years at the time of enrollment; and (c) a period of at least 1 year off‐therapy. Exclusion criteria were as follows: (a) surgical removal of the tumor without radiation and/or chemotherapy.

#### 2.1.2 Controls

The control group comprised healthy children and adolescents matched for age and sex with the non‐CNS CC survivors. They were recruited among siblings of the non‐CNS CC survivors, via advertisements disseminated through the hospital intranet, and via self‐created flyers displayed in the hospital and its neighborhood. Inclusion criteria for healthy control subjects were as follows: (a) aged 7–16 years; (b) no chronic illness potentially influencing development (e.g., birth deformity, congenital heart defects, cerebral palsy, or epilepsy); (c) no medical problems potentially influencing development (e.g., meningitis, encephalopathy, or traumatic brain injury); (d) no pervasive developmental disorders (e.g., autism); and (e) normal or corrected‐to‐normal vision and hearing.

An invitation letter with an information brochure was sent to all the participants. After approximately 2 weeks, participants and caregivers were contacted by telephone. A standardized screening telephone interview was conducted to clarify whether the participants met the inclusion criteria. All the participants and their caregivers—if participants were aged <14 years—provided written informed consent, in accordance with the Code of Ethics of the World Medical Association (Declaration of Helsinki).

### 2.2 Cognitive assessment

All the children included in the study underwent a comprehensive cognitive assessment at the Division of Neuropediatrics, Development and Rehabilitation at the University Hospital in Bern, Switzerland. The cognitive assessment took about 90 min, including a short break after 45 min. The assessments were conducted by psychologists, and by postgraduate students and study assistants under supervision. A detailed description of the assessment has been published in the study protocol (Benzing et al., 2018). For the present study, the following variables were assessed:

#### 2.2.1 Processing speed

Processing speed was assessed with the processing speed index of the Wechsler Intelligence Scale for Children (WISC‐IV; Wechsler, 2003). This index includes two subtests, namely symbol search and coding. In the symbol search subtest, the child is required to mark whether or not certain target symbols appear in a given row of symbols. In the coding subtest, the child is required to copy symbols that are paired with numbers using a key as quickly as possible. Both subtests are time‐limited to 120 s and the number of symbols correctly identified in the given time is counted. Raw scores are transformed into scaled scores for each subtest, and further transformed into standard scores for the processing speed index. Due to incompliance, information on processing speed is missing for one patient.

#### 2.2.2 Selective attention

Selective attention was assessed with the cancellation subtest of the WISC‐IV (Wechsler, 2003). The child is required to look at a random sequence of pictures and to mark certain target pictures. This is a time‐limited test and the number of target pictures correctly marked within the specified time frame is counted. Raw scores are transformed into scaled scores.

#### 2.2.3 Executive functions

Executive functions were operationalized with three constructs ( inhibition, shifting, and working memory), which were summed into a composite score for executive function. *Inhibition* was assessed with the third condition of the color‐word interference subtest of the Delis‐Kaplan Executive Function System (D‐KEFS; Delis et al., 2001). Under this condition, the child is required to name the color in which the word is written, but not to read out the word itself. The dependent variable represents the time taken to complete the task. *Shifting* was measured with the fourth condition of the color‐word interference subtest of the D‐KEFS (Delis et al., 2001). This requires the child to switch between reading out the color word, when the word is encircled by a rectangle, and naming the color of the word, when the word is not encircled by a rectangle. The dependent variable was the time needed to complete the fourth condition.

*Working memory* was assessed with the Block Recall subtest of the Working Memory Test Battery for Children (WMTB‐C; Pickering & Gathercole, 2001). This is a Corsi block task consisting of nine blocks marked with numbers visible only to the experimenter. The experimenter taps a sequence of blocks and the child is required to tap the blocks in exactly the same order as demonstrated by the experimenter (only Corsi forward spans). The dependent variable was the score of correct trials. Raw scores were transformed into standard scores for all executive functions.

Due to the restricted age range of the normative values of the D‐KEFS and the WMTB‐C, D‐KEFS scores are missing in children <8 years (*n* = 4 patients; *n* = 5 controls) and WMTB‐C scores are missing in adolescents >16 years (*n* = 1 control).

#### 2.2.4 Memory

Verbal memory was operationalized with the subtest “Atlantis Delayed” of the Kaufman Assessment Battery for Children (K‐ABC; Melchers & Preuß, 2003). In this task, the experimenter teaches the nonsense names for pictures of plants, shells, and fish. When the experimenter reads out one of the nonsense names, the child is required to point to the correct picture. Then, 15–25 min later, the assessor reads the nonsense names for pictures of plants, shells, and fish once more and the child has to point to the correct picture again. For the variable verbal memory, we only took the scores from the delayed recall without controlling for the initial recall. Raw scores were transformed into standard scores.

#### 2.2.5 Conceptual reasoning

Conceptual reasoning was assessed with the Test of Non‐Verbal Intelligence (TONI‐4; Brown et al., 2010). In this test, the experimenter shows the child an array of pictures, which contains an empty rectangle. The child is required to choose the correct picture out of a selection of various pictures. The number of correctly solved tasks was transformed into age‐based standard scores.

#### 2.2.6 S**ocioeconomic status**

The socioeconomic status (SES) was assessed using the family affluence scale (FAS; Boudreau & Poulin, 2009). Scores ranged from zero to nine with higher scores representing higher SES. Due to a lack of return of parental questionnaires, some participants miss on information on the SES (*n* = 4 controls, *n* = 2 patients).

### 2.3 Neuroimaging

#### 2.3.1 Image acquisition and data analysis

The MRI examination took place at the Institute of Diagnostic and Interventional Neuroradiology at the University Hospital of Bern. Magnetic resonance images were obtained using a 3‐Tesla Siemens Magnetom Prisma VE11C Scanner (Siemens Erlangen) equipped with a 64‐channel head coil. T1‐weighted structural brain images (acquisition time, TA = 4:33 min; repetition time, TR = 1,950 ms; echo time, TE = 2.19 ms; slices per slap = 176; field of view, FoV = 256 × 256; isovoxel resolution = 1 mm^3^) were obtained using a 3‐D T1 magnetization‐prepared rapid gradient echo sequence. Functional imaging was performed using a multiband echo‐planar imaging sequence (Release 014, VE11C) from the University of Minnesota (Center for Magnetic Resonance Research), with a distance factor of 0% (gap 0 mm), excitation pulse duration of 5,120 μs, flip angle of 30° (avoiding rf‐clipping; is in the order of the Ernst angle for repetition time (TR = 300 ms) and T1 of gray matter), multiband factor (S) = 8, *N* = 32 slices, and TA = 5:06 min. Each scan consisted of 1,000 image volumes. To reduce head motion, a head support system consisting of two fixation pillows, one on either side of the cheeks, was used. Earplugs reduced the scanner noise. All participants were instructed to close their eyes, stay awake, and remain as motionless as possible. The resting‐state sequence lasted 5.06 min. In total, the MRI examination lasted approximately 1 hr including preparation and instructions. All MRIs were checked by a neuroradiologist (N.S.) for lesions and were diagnosed according to the standard setting in clinical research.

#### 2.3.2 Preprocessing

For preprocessing and statistical analysis, SPM12 software (http://www.fil.ion.ucl.ac.uk/spm/), running in a MATLAB R2013a environment (Mathworks Inc.), and additional functions from AFNI (https://afni.nimh.nih.gov/) were used. Functional images were realigned, de‐spiked (with the AFNI 3D despike function), unwarped, and corrected for geometric distortions, using the field map of each participant and slice time. The high‐resolution structural T1‐weighted image of each participant was processed and normalized using the CAT12 toolbox (http://dbm.neuro.uni‐jena.de/cat) with default settings. Each structural image was segmented into gray matter, white matter, and CSF and was denoised. Then each image was warped into MNI space by registering it to the DARTEL default template provided by the CAT12 toolbox, via the high‐dimensional DARTEL (Ashburner, 2007) registration algorithm. Finally, a skull‐stripped version of each image in native space was created. To normalize functional images into MNI space, they were co‐registered to the skull‐stripped structural image, and the parameters from the DARTEL registration were used to warp the images, which were resampled to 3 × 3 × 3 mm voxels and smoothed with an 8 mm FWHM Gaussian kernel. Since head movements have a marked effect on results, we checked all resting‐state fMRI data for motion artifacts after realignment. Resting‐state data were further preprocessed with the CONN‐fMRI toolbox (17. f). Using the CompCor method, sources of physiological and movement noise were removed (Behzadi et al., 2007). Resting‐state data were band‐pass filtered at 0.008–0.09 Hz. The preprocessing procedures can dramatically reduce movement noise and noise from non‐neural sources (Whitfield‐Gabrieli & Nieto‐Castanon, 2012).

#### 2.3.3 Functional connectivity

We chose a seed‐based approach that includes the selection of regions of interest (ROIs) and the correlation of the average BOLD time course of voxels within the ROIs with each other and with the time courses of all other voxels in the brain. A threshold of *p* < .05 is determined to identify voxels significantly correlated with the ROIs. The drawback of this approach is the requirement of a priori selection of ROIs (Lee et al., 2013). Compared with seed‐based methods, the independent component analysis (ICA) requires only few a priori assumptions but asks for a manual selection of the components of interest while distinguishing noise from physiologic signals. Despite the differences in the two approaches, Rosazza et al. (2012) showed that the results of seed‐based analysis and ICA are significantly similar in a healthy adults.

For the seed‐based analysis, we used the CONN‐fMRI toolbox (17. F; www.nitrc.org/projects/conn, RRID: SCR_009550) in SPM 12 (Whitfield‐Gabrieli & Nieto‐Castanon, 2012). Our seeds were the extracted regions of interest (ROIs) including 91 cortical areas and 15 subcortical areas from the FSL Harvard‐Oxford Atlas and 26 cerebellar areas from the automated anatomical labeling atlas (Tzourio‐Mazoyer et al., 2002). In addition, seed regions characterizing networks (i.e., the default mode, dorsal attention, and salience network) were also included. For additional analysis (i.e., association with cognitive performance, demographic, and clinical characteristics), however, only those functional ROI‐to‐ROI connections that significantly differed between patients and controls (i.e., those that persisted after multiple comparison correction), were selected. ROI‐based analysis was carried out by calculating bivariate temporal correlation of BOLD signals (time‐series) between each pair of ROIs reflecting functional connections. Bivariate correlations were then transformed into Fisher's *Z* correlation coefficients. In the first‐level connectivity analyses, ROI‐to‐ROI connectivity matrices for each pair of sources (i.e., ROIs) were computed for all the subjects. At the second level, between‐group analyses were performed using a two‐sample *t* test to determine significant differences in ROI‐to‐ROI connections (regions and regions from networks) between patients and controls using a threshold set at *p* < .05 and the false discovery rate for multiple comparisons correction.

### 2.4 Statistical analyses of cognitive variables

The Statistical Package for Social Sciences (SPSS), version 25, was used for data analysis of cognitive data. Raw scores of cognitive tests were converted into age‐normed scores. The normality of the data was analyzed with the Shapiro–Wilk test. Group differences were calculated using the two‐sample *t* test, or the Mann–Whitney *U* test when data were not normally distributed. For the subgroup analyses in respect to therapy effects (non‐CNS‐directed therapy, CNS‐directed therapy, and healthy controls), group interactions for cognitive measures were assessed with Kruskal–Wallis test. For the comparison of cognitive measures across the three subgroups, post hoc pairwise analyses (Dwass–Steel–Critchlow–Fligner test) were performed. To analyze differences in connectivity across the three subgroups, two‐sample *t* tests were conducted. The significance level was set to .05. Because all analyses were performed after the definition of a primary hypothesis and were of exploratory nature—both issues that justify to not correct for multiple testing (Althouse, 2016)‐‐, we decided to present uncorrected data. To be maximally informative, we still mention the results after corrections for multiple comparison. Multiple comparison corrections were made using the Benjamini‐Hochberg procedure (Benjamini & Hochberg, 1995). Pearson's Chi‐square tests were conducted for each variable to determine whether the number of children with impaired performance (scaled score < 7; standard score < 85) differed between groups. The association of age‐corrected cognitive variables and clinical data with functional correlation coefficients (Fisher's *Z*) was examined using Spearman's correlations. When examining the association between functional connectivity and age at diagnosis, time since treatment and treatment duration, partial correlations, controlling for age at assessment, were conducted. All of these correlation analyses were confined to those brain regions that significantly differed between patients and controls.

## 3 RESULTS

### 3.1 Study sample

Forty‐three patients and 43 controls were included in the study. Demographic and clinical data of the participants are summarized in Table 1, for demographic and clinical data in respect to CNS‐ versus non‐CNS‐directed therapy see Table S1 . No statistically significant differences were seen across the groups in terms of sex, age, or socioeconomic status. There was a significant difference in treatment duration with survivors after CNS‐directed therapy showing longer treatment duration than survivors after non‐CNS‐directed therapy.

**TABLE 1:** Demographic and clinical data of patients and controls

Variables	Patients (*n* = 43)	Controls (*n* = 43)	Test statistics^1^			Effect size^2^
			*χ* ^2^	*U*	*p* value	*r*
Sex						
Female	18 (41.9%)	19 (44.2%)	0.05		.828	
Male	25 (58.1%)	24 (55.8%)				
Age at assessment^3^ (years)						
Mean (*SD*)	10.82 (2.16)	10.52 (2.74)	789.00		.242	−.13
Range	7.92–15.05	7.08–16.17				
Socioeconomic status^4^						
Mean (*SD*)	6.90 (1.30)	6.69 (1.54)	744.00		.585	−.06
Range	5.00–9.00	4.00–9.00				
Age at diagnosis (years)						
Mean (*SD*)	4.89 (3.32)					
Range	0.62–12.74					
Time since treatment (years)						
Mean (*SD*)	4.28 (2.03)					
Range	1.10–8.80					
Treatment duration (years)						
Mean (*SD*)	1.48 (0.90)					
Range	0.10–3.20					
Tumor type *n* (%)						
Leukemia	27 (62.8%)					
Lymphoma	4 (9.3%)					
Neuroblastoma	1 (2.3%)					
Renal tumor	6 (14%)					
Soft tissue sarcoma	3 (7%)					
Germ cell tumor	1 (2.3%)					
Other malignant neoplasms and melanomas	1 (2.3%)					
Treatment *n* (%)						
Radiation therapy only	0 (0%)					
Chemotherapy only	25 (58.1%)					
Surgery, radiation therapy	1 (2.3%)^a^					
Surgery, chemotherapy	13 (30.2%)					
Radiation therapy, chemotherapy	0 (0%)					
Surgery, chemotherapy, radiation therapy	4 (9.3%)^b^					

Abbreviations: *n*, sample size; N/A, non‐applicable; *SD*, standard deviation; SES, socioeconomic status.

^1^ Pearson's Chi‐square test for frequencies and Mann–Whitney *U* test for continuous variables.

^2^ *R* = *Z*/√*N*.

^3^ Median age of patients: 10.60 years; median age of controls: 11.30 years.

^4^ Median socioeconomic status of patients: 7.00; median SES of controls: 7.00; information missing on socioeconomic status (*n* = 4 controls, *n* = 2 patients).

^a^ *n* = 1 patient with scattered radiation due to expansive preauricular alveolar rhabdomyosarcoma.

^b^ *n* = 3 nephroblastoma; *n* = 1 azinic cell carcinoma of parotid gland.

### 3.2 Cognitive assessment

Details on the cognitive performance of patients and controls are provided in Table 2. For all the tests, group mean scores of patients and controls were within the age‐appropriate range. Patients showed weaker executive functions than controls (*p*s < .05). Even after correction for multiple testing, this effect remained statistically significant (*p*s < .05). There were no significant differences in the number of participants with cognitive impairments (performance of <1 *SD* below the mean) between the patient and control group for any cognitive variable.

**TABLE 2:** Cognitive performance of patients and controls, effect sizes, and percentages of individuals with impairments

	Patients (*n* = 43)	Controls (*n* = 43)	Test statistics			Effect size	Patients *n* (%)	Controls *n* (%)	Test statistics	
			*T* (*df*)	*U*	*p*	Cohen's *d* (*r*)			*χ* ^2^	*p*
Nonverba IQ^a^										
Mean	106.77	105.81	0.383 (84)		.352	0.08	1 (2%)	0 (0%)	1.012	.314
*SD*	10.78	12.25								
Range	82–129	85–132								
Processing speed^a^, ^b^										
Mean	105.57	105.21	0.488 (82)		.314	0.03	2 (5%)	3 (7%)	0.165	.684
*SD*	13.69	13.10								
Range	81.00–131.00	79.00–134.00								
Attention^c^, ^d^										
Mean	9.91	10.26		828.50	.202	0.09	2 (5%)	6 (14%)	2.205	.138
*SD*	2.47	2.90								
Range	4.00–15.00	4.00–16.00								
Executive function^c^, ^d^										
Mean	10.42	11.52		589.50	.003*	0.31	2 (5%)	2 (5%)	0.001	.981
*SD*	1.87	2.12								
Range	4.73–14.67	5.00–14.80								
Verbal memory^c^										
Mean	11.64	12.26		782.00	.107	0.27	1 (2%)	0 (0%) 0	1.012	.314
*SD*	2.33	2.36								
Range	5.00–16.50	9.50–17.50								

Note

Patients: median attention = 10.00; median executive function = 10.27; median verbal memory = 11.50.

Controls: median attention = 11.00; median executive function = 11.07; median verbal memory = 12.50

Abbreviations: IQ, intelligence quotient; N/A, non‐applicable; *SD*, standard deviation.

^a^ Standard scores (*M* = 100, *SD* = 15).

^b^ Information missing for processing speed (*n* = 1 patient).

^c^ Scaled scores (*M* = 10, *SD* = 3).

^d^ Information missing for the D‐KEFS scores in children under 8 years (*n* = 4 patients; *n* = 5 controls) and for the WMTB‐C score in adolescents > 16 years (*n* = 1 control) as there were no normative data available for this age category.

* Significance (*p* < .05; two‐tailed).

Survivors after CNS‐directed therapy showed significant worse executive functions than the control group whereas survivors after non‐CNS‐directed therapy did not differ from controls. There were no significant differences across groups in other cognitive variables (for details on subgroup analysis see Table S1).

### 3.3 Functional connectivity

**FIGURE 1:**
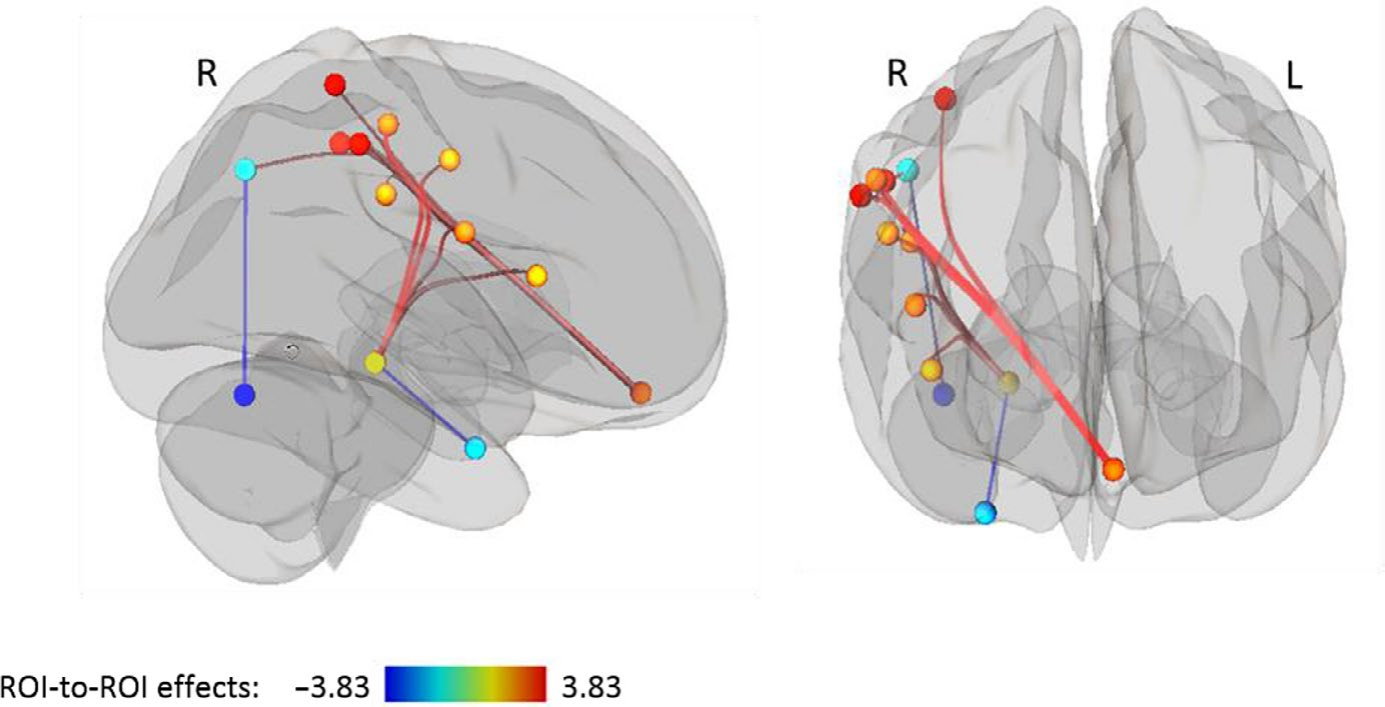
Between‐group differences in region‐to‐region functional connectivity for the contrast “patients > controls”,*p* < .05 (FDR‐corrected), two‐sided. Red lines indicate stronger functional connectivity in patients than controls. Bluelinesindicate weaker functional connectivity in patients than controls. FDR, false discovery rate; L, left hemisphere; R, right hemisphere (using radiologists' convention)

Group differences in functional connectivity between patients and controls are summarized in Table 3 (separately for regions, and for networks). No significant between‐group differences in interhemispheric connections were found; the only significant group differences were seen in intrahemispheric connections within the right hemisphere. In particular, we observed both significantly stronger and significantly weaker functional connections in the right hemisphere in patients than controls (see Figure 1). The findings for the specific regions were as follows: Patients showed significantly stronger functional connectivity between the frontal‐medial cortex and the operculum and between the frontal‐medial cortex and the supramarginal gyrus. Additionally, stronger connectivity was observed between the parahippocampal gyrus and the supramarginal gyrus and between the insula and the operculum. Weaker functional connectivity was, however, observed between the parahippocampal gyrus and the fusiform gyrus.

**TABLE 3:** Group differences in functional connectivity between patients and controls (separated for regions and networks)

Regions	Statistic *T* (*84*)	*p* value (FDR‐corrected)
Functionally stronger connections in patients than controls (P > C)		
Frontal‐medial cortex		
Parietal operculum right	−3.75	.043
Supramarginal gyrus (posterior right)	−3.49	.043
Supramarginal gyrus (anterior right)	−3.40	.043
Parahippocampal gyrus (posterior right)		
Supramarginal gyrus (anterior right)	−3.38	.039
Insular gyrus right	−3.38	.039
Central opercular cortex right	−3.27	.043
Parietal operculum right	−3.20	.046
Functionally weaker connections in patients than controls (P < C)		
Parahippocampal gyrus (posterior right)		
Fusiform gyrus (anterior right)	−3.36	.039

Networks	Statistic *T* (84)	*p* value (FDR‐corrected)
Functionally stronger connections in patients compared with controls (P > C)		
Frontal‐medial cortex		
Salience network (SMG) right	−3.38	.043
Attention network (dIPS) right	−3.32	.043
Default mode network LP right		
Salience network SMG right	−3.63	.040
Parahippocampal gyrus (posterior right)		
Salience network insula right	−3.36	.039
Sensorimotor network lateral right	−3.35	.039
Functionally weaker connections in patients than controls (P < C)		
Default mode network LP right		
Cerebellum right	3.83	.040

Note

We use radiologic convention for the presentation of left and right.

Abbreviations: C, controls; dIPS, dorsal intraparietal sulcus; FDR, false discovery rate; LP, lateral parietal; P, patients; SMG, supramarginal gyrus.

Examination of regions of networks showed that functional connectivity between the frontal‐medial cortex and the supramarginal gyrus (region of the salience network) and between the frontal‐medial cortex and the intraparietal sulcus (region of the dorsal attention network) was stronger in patients than controls. Additionally, patients showed significantly stronger functional connectivity between the lateral parietal area (region of the default mode network) and the supramarginal gyrus (region of the salience network). Stronger connectivity in patients than controls was also observed between the parahippocampal gyrus and the insula (region of the salience network) and the lateral region of the sensorimotor network. In contrast, weaker connectivity in patients than controls was observed between the lateral parietal region of the default mode network and the cerebellum.

### 3.4 Functional connectivity across subgroups

Subgroup differences between healthy controls and survivors after CNS‐ versus non‐CNS‐directed therapy are presented in Table S2. There were both, stronger and weaker functional connections in healthy controls than survivors after non‐CNS‐directed therapy. Similarly, healthy controls showed both, stronger and weaker functional connections than survivors after CNS‐directed therapy. Finally, survivors after non‐CNS‐directed therapy showed weaker functional connectivity than survivors after CNS‐directed therapy in areas included in the limbic system.

### 3.5 Association between functional connectivity and cognitive performance

**FIGURE 2:**
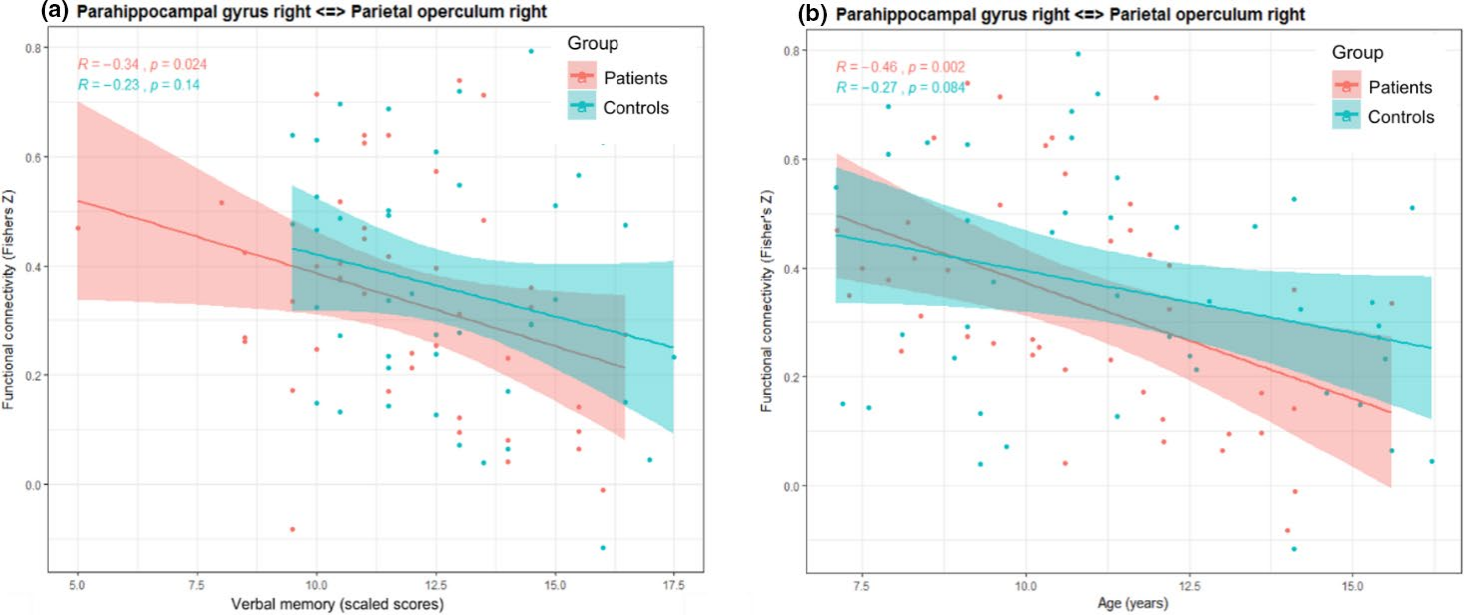
Significant correlations between functional connectivity and verbal memoryand between functional connectivity and age in patients. Verbal memory (panel a) and age (panel b) are represented on the*x*‐axis. Region‐to‐region functional connectivities (Fisher's*Z*correlation coefficients) are represented on the*y*‐axis. Panel (a): Relationship between verbal memory and functional connectivity between the right parahippocampal gyrus and the right parietal operculuminpatients (red) and controls (blue). Panel (b): Relationship between age and functional connectivity between the right parahippocampal gyrus and the right parietal operculuminpatients (red) and controls (blue). The red and blue lines represent the linear fits,the shaded areas represent the 95% CIs

**FIGURE 3:**
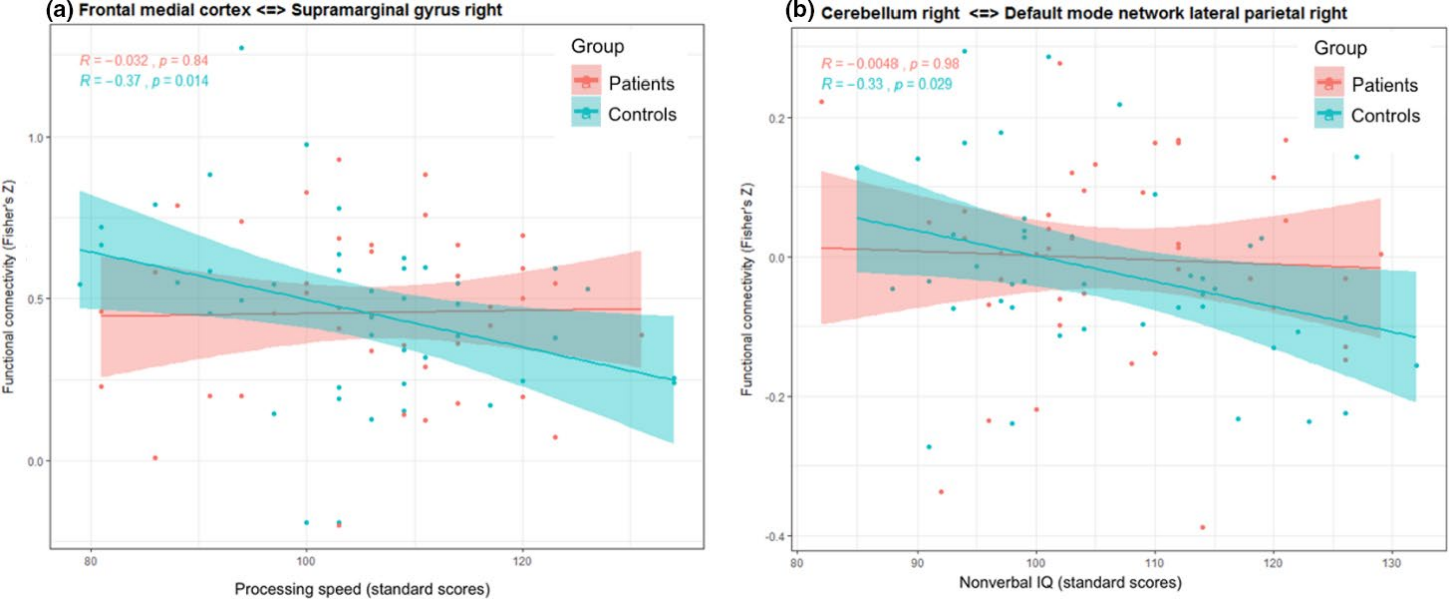
Significant correlations between functional connectivity and cognitiveperformancein controls. Processing speed (panel a) and nonverbal intelligence(IQ; panel b) are represented on the*x*‐axis. Region‐to‐region functional connectivities (Fisher's*Z*correlation coefficients) are represented on the*y*‐axis. Panel (a): Relationship between processing speed and functional connectivity between the frontal‐medial cortex and the right supramarginal gyrus across patients (red) and controls (blue). Panel (b): Relationship between nonverbal IQ and functional connectivity between the default mode network (lateral parietal area) and the cerebellum across patients (red) and controls (blue). The red and blue lines represent the linear fits and the shaded regions illustrate the 95% CIs

In the first step, associations between functional connectivity and cognitive performance were determined in regions that revealed stronger functional connectivity in patients than controls. See Figures 2and 3 and Table S3for significant associations between functional connectivity and cognitive outcome. In patients, stronger connectivity between the right parahippocampal gyrus and the right operculum was related to weaker verbal memory (*r*s (41) = −.34, *p* = .024; Figure 2a). In controls, stronger connectivity between the frontal‐medial cortex and the right supramarginal gyrus was related to slower processing speed (*r*s (41) = −0.37, *p* = .014; Figure 3a).

In the second step, associations between functional connectivity and cognitive outcome were determined in regions where functional connectivity was weaker in patients than controls. In patients, no significant associations between functional connectivity and cognitive outcome were observed. In controls, lower connectivity between the default mode network (lateral parietal) and the cerebellum was associated with better nonverbal IQ (*r*s (41) = −.33; *p* = .029; Figure 3b). Associations between functional connectivity and cognitive performance are listed in Table S3. Note that the associations described did not persist after corrections for multiple comparisons.

### 3.6 Association between functional connectivity and demographics

In patients, age at assessment was associated with the strength of connectivity between the parahippocampal gyrus and the operculum in the right hemisphere. Younger age at assessment was associated with stronger connectivity (*rs* (41) = −.46, *p* = .002; Figure 2b). The strength of functional connectivity was not significantly correlated with the duration of treatment, time since treatment, and age at diagnosis, after controlling for age (*rs* (41) < .024, *p*s > .071). Moreover, there was no relationship between the strength of functional connectivity and the socioeconomic status.

In controls, age at assessment was associated with the strength of connectivity between the frontal‐medial cortex and the intraparietal sulcus (region of the dorsal attention network) in the right hemisphere. Younger age at assessment was associated with stronger connectivity (*rs* (41) = −0.35, *p* = .024). There was no significant correlation between the strength of functional connectivity and the socioeconomic status (see Table S3). Once again, the significance of the associations did not persist after corrections for multiple comparisons.

## 4 DISCUSSION

### 4.1 Principal findings

The present cross‐sectional study investigated resting‐state functional connectivity and its relation with cognition in 43 survivors of non‐central nervous system cancer in childhood and 43 controls. So far, this is one of the first studies to investigate functional resting‐state networks in survivors of non‐CNS CC years after the termination of cancer treatment. It was hypothesized that rs‐fMRI networks in survivors of non‐CNS CC demonstrate an altered pattern of functional connectivity compared with controls. We observed significantly lower executive functions in patients than controls. More precisely, children and adolescents that were treated with CNS‐directed therapy showed significantly lower executive functions than controls, while children that were treated with non‐NS‐directed therapy showed no significant difference in executive functions compared to controls. In addition, both, stronger and weaker functional connectivity patterns occurred in patients than in controls. This finding is consistent with results of a previous rs‐fMRI study showing both hyper‐ and hypoconnectivity in 15 survivors of ALL aged 8–15 years and off‐therapy since at least 6 months (Kesler et al., 2014). Stronger as well as weaker connectivity was also described in studies with other patients, such as i.e. preterm‐born children at school‐age (Finke et al., 2015; Wehrle et al., 2018), children with ADHD (Jiang et al., 2019), or adults after traumatic brain injury (Hillary et al., 2011).

### 4.2 Differences in functional connectivity between patients and controls

Our most striking finding, the fronto‐parietal hyperconnectivity (namely the connection between the frontal‐medial cortex and the parietal operculum/supramarginal gyrus) in patients is consistent with the assumption that long‐range connections are more likely to be altered in non‐CNS CC survivors than short‐range connections (see Introduction). The frontal and parietal regions of the brain mature late in childhood and adolescence (Sowell et al., 2003). The fronto‐parietal pathway is linked to late maturing executive functions such as working memory (Østby et al., 2011)—a cognitive domain which was significantly lower in patients than controls in our sample, in particular after CNS‐directed therapy. Cortical thickness of the supramarginal gyrus and the rostral middle frontal cortex is known to be negatively associated with working memory performance in healthy children (Østby et al., 2011), suggesting that these cortical regions are important for working memory performance during childhood and adolescence. However, despite alterations in the fronto‐parietal pathway and lower executive functions in our patients’ sample, survivors still performed within the age‐appropriate range.

Beside the fronto‐parietal hyperconnectivity, we also found stronger functional connectivity pathways from the parahippocampal gyrus to parietal and temporal brain regions. There is evidence that cancer treatments have a pronounced effect on neurogenesis by reducing the amount of immature neurons. More precisely, research has shown that immature neurons were particularly reduced in the hippocampal gyri after cancer treatment (Monje et al., 2007) and cortical thickness of the parahippocampal area was lower in survivors of childhood sarcoma treated with high‐dose chemotherapy (Sleurs et al. 2020), interestingly only when the effect was uncorrected for depression. In addition, cancer treatments are known to damage oligodendrocyte precursor cells that are responsible for white matter myelination. Although studies were centered on the neurotoxic effect on the hippocampal gyri, we assume that the same mechanisms may apply for the parahippocampal gyri. Thus, the parahippocampal gyrus might be a brain region that is particularly vulnerable to the effects of pediatric cancer and its treatment. Disturbances in this susceptible brain region are likely to affect its connections to other brain regions, reflected by altered resting‐state connectivity.

Stronger functional connectivity pathways might indicate a cerebral marker that operates on an “all hands on deck” approach (Chen et al., 2016). According to the “cortical inefficiency model” (Manoach, 2003), additional resource allocation is required in order to maintain task performance in case of altered brain development. To maintain normal working memory performance, patients may need to recruit more neuronal resources (i.e., oxygen; Robinson et al., 2010). This interpretation is in line with the findings reported by Robinson et al., that is, greater working memory‐related activation occurs during a working memory task in survivors of ALL than in controls (Robinson et al., 2010). Additionally, stronger functional connectivity has been reported in adult survivors of childhood brain tumors and in adult patients with other neurological diseases (Audoin et al., 2003; Chen et al., 2016; Staffen et al., 2002; Sweet et al., 2006). Overall, the impact of increased connectivity strength has been discussed in various ways, including possible adaption mechanism employed by the brain to cope with early disease or neurotoxicity (Wehrle et al., 2018). Our findings of hyperconnectivity in survivors after CNS‐directed therapy (when compared to non‐CNS‐directed therapy) support the model of adaptation mechanisms of the brain to early events such as CC.

### 4.3 Relevance of the right hemisphere

Strikingly, in our patients, functional connectivity alterations occurred exclusively in the right hemisphere. Altered clustering in a subnetwork of the fronto‐parietal and temporal regions was similarly shown by Kesler et al. (2016) in ALL survivors, however, alterations in ALL survivors were left‐lateralized (Kesler et al., 2016). Also in a study with adult survivors of childhood ALL, the left hemisphere was suggested to be more affected than the right hemisphere (Billiet et al. 2018). What possible explanations exist for the right‐lateralized alterations we have found in our sample? Cognitive domains predominantly associated with right‐hemisphere lateralization (i.e., visuospatial, visual‐perceptual, and attentional processing tasks) are suggested to be more susceptible to alteration by irradiation and/or chemotherapy than cognitive domains typically associated with left‐hemisphere lateralization (Buono et al., 1998; Moore, 2005). This might be due to the higher proportion of white matter in the right than the left hemisphere or due to variations in hemispheric asymmetry that occur during brain maturation (Toga & Thompson, 2003). A further explanation might be the greater vulnerability in terms of blood supply of the left than the right hemisphere (Njiokiktjien, 2006). This is observed in diseases such as unilateral stroke or unilateral epilepsy, which occur more frequently in the left than the right hemisphere (Njiokiktjien, 2006). The right hemisphere might compensate for the greater vulnerability of the left hemisphere, by altering the level of functional connectivity. Alternatively, the right hemisphere might play a particularly important role in new task demands (Hillary et al., 2011) or maturational effects might play a role in determining the susceptibility of the right or left hemisphere. ALL survivors treated at a younger age (≤36 months) demonstrated right‐hemisphere dysfunctions, while those treated at older ages demonstrated left‐hemisphere dysfunctions (Waber et al., 1992). In our patients, cognitive functions typically associated with right‐hemisphere lateralization such as attention were intact and within the age‐appropriate range. Our patients—in particular survivors after CNS‐directed therapy—differ from controls in regard to executive functions, a cognitive domain which is not strictly lateralized, neither to the left nor the right hemisphere (Vallesi, 2012). As mentioned above, previous studies examining structural and functional connectivity in survivors of non‐CNS CC demonstrated primarily left‐sided (Edelmann et al., 2014; Kesler et al., 2016) and bilateral alterations (Kesler et al., 2014; Morioka et al., 2013). Given these marked differences to the present study, further studies will be necessary to replicate our findings.

### 4.4 The connectivity–cognition relationship

The study's secondary aim was to gain insights into the connectivity–cognition relationship. The human brain is organized to enable efficient functional communication through the integration of widely distributed brain regions (Van den Heuvel & Sporns, 2013). In particular, hub regions (regions that are strongly interconnected) play an important role in the integration of information; they are crucial for successful cognitive functioning (Van den Heuvel & Sporns, 2013). For example, the level of efficient functional communication of frontal and parietal hub regions is related to IQ and cognitive control (Cole et al., 2012; Van den Heuvel et al., 2009). It is assumed that cognitive improvement is driven by either an increase or decrease in functional connectivity. Implying a “less is more” hypothesis, weaker functional connectivity is related to better cognitive performance (Stevens & Spreng, 2014) whereas stronger functional connectivity might be linked to weaker cognitive performance, likely entailing compensational mechanisms that require stronger functional connectivity.

Our patients had stronger functional connectivity than our controls between the parahippocampal gyrus (serving spatial memory processes, item recognition, and the maintenance of information in working memory; Raslau et al., 2015) and the operculum (contributing to sensorimotor integration; Eickhoff et al., 2010). Weaker performance in verbal memory tests was related to stronger functional connectivity, however. After controlling for age, effects disappeared, indicating a strong influence of age on the connectivity between the parahippocampal gyrus and the parietal operculum in 7–16‐year‐olds. This finding points towards a stronger influence of age than of the cancer itself on functional connectivity at rest.

Children in the control group showed a significant negative relationship between processing speed and the functional connectivity strength between the frontal‐medial cortex and the right supramarginal gyrus. This finding fits with the “less is more” hypothesis. Conversely, better IQ was linked to a stronger connectivity between the right cerebellum and the lateral parietal part of the default mode network in controls. This is in line with the findings of other imaging studies in the context of global connectivity efficiency and intellectual functioning (van den Heuvel et al., 2009).

The question of how cognitive performance is related to functional connectivity in non‐CNS CC patients and controls is particularly difficult to answer because positive and negative associations might reflect different maturational timelines of the distinct brain regions . In our patients, age‐related changes (focusing on regions that differed between groups) were found in the functional connectivity between the parahippocampal gyrus and the operculum, which decreased with increasing age. In controls, the same pattern was observed, but the relationship was not statistically significant.

### 4.5 Limitations

Certain limitations apply to this study. Firstly, survivors of non‐CNS CC represent a heterogeneous population with different cancer types and treatment protocols. Given that CNS‐directed therapy is associated with a higher degree of neurotoxicity, we differentiated the heterogenous sample into survivors of non‐CNS CC. Furthermore, we did not distinguish the deleterious effects of cancer from the potential deleterious effects of treatment protocols. This could be done using a longitudinal serial neuroimaging study approach to examine within‐person changes before and after cancer treatment.

Secondly, our correlational analyses examining the relationship between functional connectivity and cognition were performed after the definition of a primary hypothesis and were of exploratory nature—both issues that justify to not corrected for multiple testing (Althouse, 2016). Thirdly, we used a seed‐based approach that conducts bivariate correlation analysis of two regions of interest and represents one of the oldest and most straightforward functional connectivity approaches. A clear advantage of seed‐based approaches is that they can address specific questions regarding the brain connectivity pattern between two regions. A disadvantage, however, which results from correlating “only” the temporal signals of one system (one seed region) with the whole brain, is that other signals (i.e., networks) occurring simultaneously are disregarded. This may lead to an under‐representation of the developmental dynamics of the given network data (Bijsterbosch, 2017; Cole et al., 2010). Graph theory, for example, might overcome this disadvantage by providing multiple measures of network properties (Lee et al., 2013). Last but not least, since the literature on alterations in resting‐state connectivity after non‐CNS CC is scarce, we were unable to choose specific ROIs a priori from the default predefined ROIs, but instead had to include all implemented ROIs.

## 5 CONCLUSION

The findings of the present study indicate that, even in the absence of brain lesions, CC and its treatment can significantly affect the developing brain years after cancer and its treatment. Alterations along the cortico‐cortical and cortico‐subcortical pathways occur in the right hemisphere, which seems to be particularly susceptible to non‐CNS CC. Hyperconnectivity was associated with worse verbal memory. Our findings underscore the need for monitoring cognitive deveopment in survivors of non‐CNS CC even years after cancer and its treatment, in particular in children and adolescents treated with CNS‐directed therapy. The examination of the complex question regarding the impact of non‐CNS CC and its treatments on the developing brain has only recently begun. Future multimodal studies that combine various neuro imaging techniques with specific cognitive assessments are required to obtain the full picture of the neural effects of non‐CNS CC and its relation with cognition.

## CONFLICT OF INTEREST

All authors confirm that they do not have any conflict of interests.

## AUTHOR CONTRIBUTION

KL, CR, MS, MG, and RE involved in conception or design of the work. JS, VB, VS, CK, NS, and RE involved in data collection. JS, MK, LK, RE, MPW, and CK involved in data analysis and interpretation. JS and RE involved in drafting the article. JS, MK, LK, VB, VS, MPW, CK, NS, MG, CR, MS, KL, and RE involved in critical revision of the article. JS, MK, LK, VB, VS, MPW, CK, NS, MG, CR, MS, KL, and RE involved in final approval of the version to be published.

### Peer Review

The peer review history for this article is available at https://publons.com/publon/10.1002/brb3.1931.

## Supporting information

Supplementary Material

Supplementary Material

Supplementary Material

## Data Availability

The data that support the findings of this study are available from the corresponding author upon reasonable request.
