# Supplementary material for: Impact of non‐CNS childhood cancer on resting‐state connectivity and its association with cognition
Source: Brain Behav. 2020 Nov 18;11(1):e01931. doi: 10.1002/brb3.1931 (PMC7821559; doi:10.1002/brb3.1931)
Supplement: Supplementary file 1 — Supplementary Material [file BRB3-11-e01931-s001.docx]

Table S1.

*Cognitive performance in respect to therapy approach (non-CNS-directed therapy versus CNS-directed therapy)*

| Domain | 0  Healthy controls  (n = 43) | | 1  non-CNS-directed  therapy (n= 22) | | 2  CNS-directed therapy  (n = 21) | | 0 vs 1 | | 0 vs 2 | | 1 vs 2 | | Group  Interaction | | | |
| --- | --- | --- | --- | --- | --- | --- | --- | --- | --- | --- | --- | --- | --- | --- | --- | --- |
|  | Mean | SD | Mean | SD | Mean | SD | *W* | *p* | *W* | *p* | *W* | *p* | Chi | df | *p* | *ε^2^* |
| Age | 11.50 | 2.74 | 11.20 | 2.08 | 10.50 | 2.25 | 0.56 | .918 | 2.16 | .277 | 1.38 | .595 | 2.346 | 2 | .309 | 0.309 |
| SES | 6.69 | 1.54 | 7.00 | 1.30 | 6.80 | 1.32 | 0.92 | .794 | 0.34 | .970 | 0.61 | .904 | 0.452 | 2 | .798 | 0.006 |
| Gender | 0.44 | 0.50 | 0.55 | 0.51 | 0.29 | 0.46 | 1.11 | .713 | 1.69 | .458 | 2.41 | .203 | 2.969 | 2 | .227 | 0.034 |
| Age at diagnosis §§ | N/A | N/A | 5.29 | 3.78 | 4.45 | 2.79 | N/A | N/A | N/A | N/A | 205.0 | .536 | N/A | N/A | N/A | N/A |
| Time since treatment §§ | N/A | N/A | 5.84 | 2.48 | 5.83 | 1.44 | N/A | N/A | N/A | N/A | 211.5 | .840 | N/A | N/A | N/A | N/A |
| Treatment duration §§ | N/A | N/A | 0.89 | 0.77 | 2.10 | 0.52 | N/A | N/A | N/A | N/A | 50.5 | <.001** | N/A | N/A | N/A | N/A |
| Nonverbal IQ † | 106.00 | 12.20 | 104.00 | 11.10 | 110.00 | 9.50 | 0.84 | .822 | 2.32 | .230 | 2.81 | .116 | 4.261 | 2 | .119 | 0.050 |
| Processing speed †^,^ ‡ | 105.00 | 13.10 | 106.00 | 13.30 | 108.00 | 11.20 | 0.05 | .999 | 1.49 | .544 | 1.05 | .736 | 1.104 | 2 | .576 | 0.013 |
| Selective attention §¶ | 10.30 | 2.90 | 10.30 | 2.47 | 9.52 | 2.46 | 0.39 | .960 | 1.56 | .512 | 1.25 | .652 | 1.353 | 2 | .508 | 0.016 |
| Executive functions § | 11.50 | 2.12 | 10.60 | 2.16 | 10.30 | 1.58 | 2.45 | .194 | 3.89 | .016* | 1.10 | .715 | 8.294 | 2 | .016* | 0.099 |
| Verbal memory § | 12.30 | 2.36 | 10.80 | 2.71 | 12.30 | 1.93 | 3.09 | .074 | 0.28 | .979 | 2.97 | .089 | 5.876 | 2 | .053 | 0.069 |

Note: N/A = non-applicable; SD = standard deviation; SES = Socioeconomic status.

0 vs 1; 0 vs 2 and 1 vs 2 was analyzed using the Dwass-Steel-Critchlow-Fligner Test.

Group interactions were assessed using Mann-Whitney U-test.

§§ Mann-Whitney U-test was used for the comparison between group 1 and group 2.

* Significance (*p* < .05; two-tailed).

*p* = *p* value.

† Standard scores (*M* = 100, *SD* = 15).

‡Information missing for processing speed (n = 1 patient).

§ Scaled scores (*M* = 10, *SD* = 3).

¶ Information missing for the D-KEFS scores in children under 8 years (n = 4 patients; n = 5 controls) and for the WMTB-C score in adolescents >16 years (n = 1 control) as there were no normative data available for this age category.

Patients: median attention = 10.00; median executive function = 10.27; median verbal memory = 11.50.

Controls: median attention = 11.00; median executive function = 11.07; median verbal memory = 12.

*ε^2^* partial epsilon squared (effect size).
