# Supplementary material for: Impact of non‐CNS childhood cancer on resting‐state connectivity and its association with cognition
Source: Brain Behav. 2020 Nov 18;11(1):e01931. doi: 10.1002/brb3.1931 (PMC7821559; doi:10.1002/brb3.1931)
Supplement: Supplementary file 2 — Supplementary Material [file BRB3-11-e01931-s002.docx]

Table S2

1. *Differences in functional connectivity between healthy controls and survivors after non-CNS-directed therapy (0>1) separated for regions and networks*

| Regions | Statistic  *T* (*63*) | *p* value (FDR-corrected) |
| --- | --- | --- |
| **Functionally stronger connections in healthy controls compared to survivors after non-CNS-directed therapy** | | |
| Angular gyrus left |  |  |
| Hippocampus left | 4.43 | .006 |
| **Functionally weaker connections in healthy controls compared to survivors after non-CNS-directed therapy** | | |
| Lateral occipital cortex (inferior devision right) |  |  |
| Parietal operculum cortex left | −3.33 | .047 |
| Superior temporal gyrus (posterior division left) | −3.57 | .047 |
| Superior temporal gyrus (anterior division left) | −3.39 | .047 |
| Planum temporale left | −3.56 | .047 |
| Parahippocampal gyrus (posterior division right) |  |  |
| Supramarginal gyrus (anterior division right) | −3.72 | .030 |
| Frontal operculum cortex right | −3.55 | .030 |
| Parahippocampal gyrus (posterior division left) |  |  |
| Supramarginal gyrus (anterior division right) | −3.69 | .039 |
| Frontal pole right |  |  |
| Superior temporal gyrus (posterior division right) | −3.96 | .031 |

| Networks | Statistic  T (*63*) | *p* value (FDR-corrected) |
| --- | --- | --- |
| **Functionally stronger connections in healthy controls compared to survivors after non-CNS-directed therapy** | | |
| Hippocampus left |  |  |
| Frontoparietal network (PPC) left | 3.69 | .038 |
| Default mode network LP right |  |  |
| Cerebellum Crus (1) right | 3.84 | .047 |
| **Functionally weaker connections in healthy controls compared to survivors after non-CNS-directed therapy** | | |
| Parahippocampal gyrus (posterior division right) |  |  |
| Salience network insula right | −3.80 | .030 |
| Dorsal attention network IPS right | −3.56 | .030 |
| Lateral occipital cortex (inferior devision right) |  |  |
| Sensorimotor network lateral left | −3.44 | .047 |
| Language network IFG right |  |  |
| Inferior frontal gyrus (pars opercularis right) | −3.84 | .047 |
| Frontal medial cortex |  |  |
| Dorsal attention network IPS right | −3.83 | .049 |

1. *Differences in functional connectivity between healthy controls and CNS-directed therapy (0>2) separated for regions and networks*

| Regions | Statistic  *T* (*62*) | *p* value (FDR-corrected) |
| --- | --- | --- |
| **Functionally stronger connections in healthy controls compared to survivors after CNS-directed therapy** | | |
| Cerebellum (8) right |  |  |
| Insular cortex right | 3.34 | .039 |
| Supplementary motor cortex right | 3.35 | .039 |
| Parietal operculum right | 3.38 | .039 |
| Inferior frontal gyrus (pars opercularis) | 3.62 | .033 |
| **Functionally weaker connections in healthy controls compared to survivors after CNS-directed therapy** | | |
| Parahippocampal gyrus (anterior right) |  |  |
| Cerebellum (45) right | −3.61 | .033 |
| Hippocampus left | −3.88 | .042 |
| Putamen left | −3.41 | .048 |
| Vermis (3) | −4.15 | .017 |
| Accumbens left |  |  |
| Vermis (45) | −4.00 | .028 |

| Networks | Statistic  T (*62*) | *p* value (FDR-corrected) |
| --- | --- | --- |
| **Functionally stronger connections in healthy controls compared to survivors after CNS-directed therapy** | | |
| Cerebellum right |  |  |
| Salience network (ACC) | 4.00 | .028 |
| Language network (pSTG) right | 3.69 | .033 |
| Salience network (SMG) right | 3.26 | .043 |
| **Functionally weaker connections in healthy controls compared to survivors after CNS-directed therapy** | | |
| Frontal medial cortex |  |  |
| Salience network SMG | −3.71 | .049 |
| Frontoparietal network PPC | −3.61 | .049 |

| Regions | Statistic  *T* (*41*) | *p* value (FDR-corrected) |
| --- | --- | --- |
| **Functionally weaker connections in survivors after non-CNS-directed therapy compared to survivors after CNS-directed therapy** | | |
| Caudate left |  |  |
| Occipital pole left | −4.32 | .008 |
| Occipital pole right | −4.14 | .008 |
| Accumbens left |  |  |
| Thalamus right | −4.08 | .022 |
| Thalamus left | −3.53 | .043 |
| Vermis (45) | −3.95 | .022 |
| Cerebellum (45) left | −3.86 | .022 |
| Accumbens right |  |  |
| Middle temporal gyrus (anterior division left) | −3.96 | .047 |
| Paracingulate gyrus left |  |  |
| Occipital pole right | −4.19 | .024 |

1. *Differences in functional connectivity between survivors after non-CNS-directed therapy and survivors after CNS-directed therapy (1>2) separated for regions and networks*

| Networks | Statistic  T (*41*) | *p* value (FDR-corrected) |
| --- | --- | --- |
| **Functionally weaker connections in survivors after non-CNS-directed therapy compared to survivors after CNS-directed therapy** | | |
| Caudate left |  |  |
| Visual occipital network | −4.39 | .013 |
