# Supplementary material for: Impact of non‐CNS childhood cancer on resting‐state connectivity and its association with cognition
Source: Brain Behav. 2020 Nov 18;11(1):e01931. doi: 10.1002/brb3.1931 (PMC7821559; doi:10.1002/brb3.1931)
Supplement: Supplementary file 3 — Supplementary Material [file BRB3-11-e01931-s003.docx]

| Table S3 | | | | | | | | | | | | | | | |
| --- | --- | --- | --- | --- | --- | --- | --- | --- | --- | --- | --- | --- | --- | --- | --- |
| 1. Associations between Functional Connectivity and Cognitive as well as Demographic Parameters in Patients (networks in brackets) | | | | | | | | | | | | | | | |
|  | FMC-  Op | FMC-SMGp | FMC-SMGa | FMC-SMG  (SN) | FMC-dIPS  (AN) | (DMN)-SMGr  (SN) | Cb-  (DMN) | PHG-  IG | PHG-SMG | PHG-FuG | PHG-  Op | PHG- POp | PHG-(SMN) | PHG-  IG  (SN) |  |
|  |  |  |  |  |  |  |  |  |  |  |  |  |  |  |  |
|  | rs | rs | rs | rs | rs | rs | rs | rs | rs | rs | rs | rs | rs | rs |  |
|  | (p) | (p) | (p) | (p) | (p) | (p) | (p) | (p) | (p) | (p) | (p) | (p) | (p) | (p) |  |
| Nonverbal IQ | -0.12 | -0.07 | -0.07 | 0.03 | -0.25 | 0.03 | -0.01 | -0.03 | -0.2 | 0.01 | -0.03 | -0.1 | 0.23 | 0.08 |  |
|  | *(.444)* | *(.660)* | *(.679)* | *(.862)* | *(.112)* | *(.870)* | *(.938)* | *(.844)* | *(.201)* | *(.943)* | *(.861)* | *(.524)* | *(.135)* | *(.625)* |  |
| Processing speed | -0.08 | 0.01 | -0.03 | -0.05 | 0.06 | -0.18 | 0.24 | -0.16 | -0.1 | **0.34** | 0.17 | -0.17 | 0.3 | 0.23 |  |
|  | *(.614)* | *(.969)* | *(.869)* | *(.772)* | *(.722)* | *(.247)* | *(.134)* | *(.317)* | *(.544)* | ***(.028*)*** | *(.289)* | *(.293)* | *(.52)* | *(.142)* |  |
| Attention | -0.06 | 0 | 0.28 | 0.08 | -0.22 | -0.01 | -0.09 | -0.2 | 0.06 | -0. 10 | -0.06 | -0.12 | 0.09 | -0.05 |  |
|  | *(.718)* | *(.993)* | *(.65)* | *(.606)* | *(.162)* | *(.959)* | *(.572)* | *(.195)* | *(.697)* | *(.507)* | *(.726)* | *(.429)* | *(.558)* | *(.751)* |  |
| Executive function | 0.02 | 0.1 | 0.11 | -0.16 | 0.02 | -0.13 | 0. 12 | -0.22 | 0.18 | 0.04 | 0.17 | 0.09 | 0.3 | 0.03 |  |
|  | *(.915)* | *(.513)* | *(.485)* | *(.307)* | *(.888)* | *(.420)* | *(.463)* | *(.157)* | *(.238)* | *(.822)* | *(.288)* | *(.561)* | *(.53)* | *(.862)* |  |
| Verbal memory | 0.06 | 0.01 | 0.2 | 0.02 | -0.04 | 0.04 | 0.23 | 0.05 | 0.06 | 0.12 | -0.05 | **-0.34** | 0.1 | 0.03 |  |
|  | *(.688)* | *(.963)* | *(.194)* | *(.887)* | *(.815)* | *(.825)* | *(.138)* | *(.738)* | *(.713)* | *(.459)* | *(.730)* | ***(.024*)*** | *(.528)* | *(.862)* |  |
| Age at assessment | 0.1 | -0.16 | 0.26 | -0.1 | -0.28 | -0.32 | 0.17 | 0.24 | 0.22 | 0.03 | -0.22 | **-0.46** | 0.23 | 0.07 |  |
|  | *(.506)* | *(.305)* | *(.940)* | *(.521)* | *(.700)* | *(.700)* | *(.279)* | *(.118)* | *(.149)* | *(.850)* | *(.155)* | ***(.002*)*** | *(.142)* | *(.643)* |  |
| SES^a^ | -0.05 | 0.07 | -0.05 | -0.01 | -0.01 | -0.05 | 0.08 | 0.07 | -0.13 | 0.07 | -0.03 | -0.04 | 0.14 | 0.26 |  |
|  | *(.777)* | *(.685)* | *(.765)* | *(.976)* | *(.977)* | *(.755)* | *(.638)* | *(.680)* | *(.421)* | *(.645)* | *(.842)* | *(.815)* | *(.391)* | *(.107)* |  |
| *Note*. rs = Spearman rho; * indicates significance (p < .05; two-tailed); ^a^ socioeconomic status.  FMC – Op: frontal medial cortex – operculum,  FMC – SMGp: frontal medial cortex - supramarginal gyrus posterior,  FMC – SMGa: frontal medial cortex - supramarginal gyms anterior,  FMC – SMG (SN): frontal medial cortex - supramarginal gyms (salience network),  FMC – dIPS (AN): frontal medial cortex - dorsal intraparietal sulcus (attention network),  (DMN) - SMGr (SN): default mode network - supramarginal gyrus (salience network),  Cb – (DMN): cerebellum - default mode network,  PHG – IG: parahippocampal gyrus - insular gyms,  PHG – SMG: parahippocampal gyrus - supramarginal gyrus,  PHG – FuG: parahippocampal gyrus – fusiform gyms,  PHG – Op: parahippocampal gyrus – operculum,  PHG – POp: parahippocampal gyrus - parietal operculum,  PHG – (SMN): parahippocampal gyrus - sensorimotor network,  PHG – IG (SN): parahippocampal gyrus - insular gyrus (salience network). | | | | | | | | | | | | | | | |

| 1. *Associations between Functional Connectivity and Cognitive as well as Demographic Parameters in Controls (networks in brackets)* | | | | | | | | | | | | | | | |
| --- | --- | --- | --- | --- | --- | --- | --- | --- | --- | --- | --- | --- | --- | --- | --- |
|  | FMC-  Op | FMC-SMGp | FMC-SMG  (SN) | FMC-SMG  (SN) | FMC-dIPS  (AN) | (DMN)-SMGr  (SN) | Cb-(DMN) | PHG-  IG | PHG-SMG | PHG-  FuG | PHG-  Op | PHG-  POp | PHG-(SMN) | PHG-  IG  (SN) |  |
|  |  |  |  |  |  |  |  |  |  |  |  |  |  |  |  |
|  | rs | rs | rs | rs | rs | rs | rs | rs | rs | rs | rs | rs | rs | rs |  |
|  | *(p)* | *(p)* | *(p)* | *(p)* | *(p)* | *(p)* | *(p)* | *(p)* | *(p)* | *(p)* | *(p)* | *(p)* | *(p)* | *(p)* |  |
| Nonverbal IQ | -0.04 | -0.02 | -0.02 | -0 .03 | -0 .08 | 0.24 | -0.33 | 0.10 | 0.08 | 0.05 | 0.00 | 0.07 | -0.04 | 0.08 |  |
|  | *(.785)* | *(.917)* | *(.894)* | *(.863)* | *(.627)* | *(.124)* | *(.029* )* | *(.532)* | *(.615)* | *(.761)* | *(.978)* | *(.657)* | *(.798)* | *(.620)* |  |
| Processing speed | -0.30 | **-0.37** | -0.06 | 0.08 | -0.14 | 0.05 | -0.25 | -0.29 | -0.09 | -0.03 | -0.00 | -0.28 | 0.16 | -0.01 |  |
|  | *(.053)* | ***(.014*)*** | *(.720)* | *(.611)* | *(.386)* | *(.729)* | *(.112)* | *(.057)* | *(.550)* | *(.871)* | *(.983)* | *(.073)* | *(.312)* | *(.976)* |  |
| Attention | -0.10 | -0.17 | -0.07 | -0 .13 | -0.01 | 0.12 | -0.16 | 0.00 | 0.11 | 0.13 | 0.11 | 0.02 | 0.21 | 0.24 |  |
|  | *(.514)* | *(.292)* | *(.663)* | *(.398)* | *(.945)* | *(.437)* | *(.299)* | *(.978)* | *(.465)* | *(.408)* | *(.483)* | *(.900)* | *(.185)* | *(.129)* |  |
| Executive function | -0.24 | -0.20 | -0.07 | 0.06 | 0.16 | 0.18 | -0.09 | -0.17 | -0.12 | 0.07 | 0.02 | -0.15 | -0 .05 | -0.04 |  |
|  | *(.119)* | *(.203)* | *(.664)* | *(.715)* | *(.321)* | *(.236)* | *(.588)* | *(.275)* | *(.445)* | *(.667)* | *(.901)* | *(.325)* | *(.773)* | *(.806)* |  |
| Verbal memory | -0.22 | -0.21 | -0.09 | 0.10 | -0.03 | 0.13 | 0.05 | -0.11 | -0.14 | 0.27 | 0.10 | -0.23 | -0 .10 | 0.06 |  |
|  | *(.163)* | *(.182)* | *(.574)* | *(.522)* | *(.855)* | *(.394)* | *(.757)* | *(.476)* | *(.364)* | *(.078)* | *(.522)* | *(.142)* | *(.526)* | *(.695)* |  |
| Age at assessment | -0.14 | -0.29 | -0.06 | -0.22 | -0.35 | -0.27 | 0.20 | -0.22 | -0.00 | -0.11 | 0.01 | -0.27 | 0.09 | 0.15 |  |
|  | *(.359)* | *(.061)* | *(.719)* | *(.154)* | *(.024*)* | *(.085)* | *(.20 7)* | *(.152)* | *(.997)* | *(.468)* | *(.971)* | *(.084)* | *(.575)* | *(.328)* |  |
| SES^a^ | 0.06 | -0.09 | 0.08 | 0.01 | -0.04 | -0. 11 | -0.03 | -0.01 | 0.16 | 0.07 | -0.08 | 0.10 | 0.31 | 0.01 |  |
|  | *(.739)* | *(.598)* | *(.634)* | *(.951)* | *(.816)* | *(.510)* | *(.867)* | *(.947)* | *(.336)* | *(.655)* | *(.611)* | *(.555)* | *(.059)* | *(.965)* |  |
| *Note.* rs = Spearman rho, * Indicates significance (p < .05; two-tailed), ^a^ socio-economic status,  FMC – Op: frontal medial cortex - operculum,  FMC – SMGp: frontal medial cortex - supramarginal gyrus posterior,  FMC – SMGa: frontal medial cortex - supramarginal gyms anterior,  FMC - SMG (SN): frontal medial cortex - supramarginal gyms (salience network),  FMC - dIPS (AN): frontal medial cortex - dorsal intraparietal sulcus (attention network),  (DMN) - SMGr (SN): default mode network - supramarginal gyrus (salience network),  Cb – (DMN): cerebellum - default mode network,  PHG – IG: parahippocampal gyrus - insular gyms,  PHG – SMG: parahippocampal gyrus - supramarginal gyrus,  PHG – FuG: parahippocampal gyrus – fusiform gyms,  PHG – Op: parahippocampal gyrus - operculum,  PHG – Pop: parahippocampal gyrus - parietal operculum,  PHG – (SMN): parahippocampal gyrus - sensorimotor network,  PHG - IG (SN): parahippocampal gyrus - insular gyrus (salience network). | | | | | | | | | | | | | | | |
